# Complement Inhibitors in Age-Related Macular Degeneration: A Potential Therapeutic Option

**DOI:** 10.1155/2021/9945725

**Published:** 2021-07-29

**Authors:** Shuqi Qin, Ning Dong, Ming Yang, Jialin Wang, Xue Feng, Yanling Wang

**Affiliations:** ^1^Department of Ophthalmology, Beijing Friendship Hospital, Capital Medical University, Beijing, China; ^2^Department of Ophthalmology, Beijing Moslem People's Hospital, Beijing, China

## Abstract

Age-related macular degeneration (AMD) is a multifactorial disease, which can culminate in irreversible vision loss and blindness in elderly. Nowadays, there is a big gap between dry AMD and wet AMD on treatment. Accounting for nearly 90% of AMD, dry AMD still lacks effective treatment. Numerous genetic and molecular researches have confirmed the significant role of the complement system in the pathogenesis of AMD, leading to a deeper exploration of complement inhibitors in the treatment of AMD. To date, at least 14 different complement inhibitors have been or are being explored in AMD in almost 40 clinical trials. While most complement inhibitors fail to treat AMD successfully, two of them are effective in inhibiting the rate of GA progression in phase II clinical trials, and both of them successfully entered phase III trials. Furthermore, recently emerging complement gene therapy and combination therapy also offer new opportunities to treat AMD in the future. In this review, we aim to introduce genetic and molecular associations between the complement system and AMD, provide the updated progress in complement inhibitors in AMD on clinical trials, and discuss the challenges and prospects of complement therapeutic strategies in AMD.

## 1. Introduction

Age-related macular degeneration (AMD) is a significant cause of irreversible blindness and vision impairment among the elderly in developed countries [1, 2]. With the transformation of the aging population, it is estimated that by 2040, there will be around 288 million AMD patients worldwide [3]. AMD is generally classified into either early, intermediate, or advanced stages, the latter of which can be further subdivided into exudative (wet) and nonexudative (dry) phenotypes [4]. More specifically, wet AMD, also known as neovascular AMD, is characterized by a rapid and substantial vision loss, which is caused by the formation of macular neovascularization. Featuring hemorrhage, edema, and scar formation of retinal tissue, advanced wet AMD occurs partly due to the upregulation of vascular endothelial growth factor (VEGF). Moreover, accounting for about 90% of AMD [5], dry AMD will give rise to macular atrophy and progressive vision loss, featuring photoreceptors, retinal pigment epithelium, and choroidal capillary degeneration, which can be referred to geographic atrophy (GA) as well [6].

The pathogenesis of AMD is complex. Although the identification of genetic risk factors for AMD has been quite successful, the mechanisms by which these risk factors interact in AMD are still unclear. Thus, the discovery of future therapeutic strategy for AMD is still full of challenges. Nowadays, there is still a big gap between dry AMD and wet AMD on treatment. On the one hand, wet AMD is currently treatable. Monthly intravitreal injections of anti-VEGF drugs (such as ranibizumab, aflibercept, and bevacizumab) have become the first-line treatment for wet AMD, which can reduce the incidence of severe vision loss significantly [7]. However, this measure is relatively effective only in a limited population. In this way, a new treatment for wet AMD is also demanded. On the other hand, although some progress has been made in the pathogenesis, there is still no approved treatment or effective therapy in dry AMD [8]. Consequently, compared with wet AMD, it is more urgent to explore an effective treatment for dry AMD.

Numerous genetic studies and molecular research have confirmed the significant role of the complement system in AMD, including genetic variants, overactivation of alternative pathway, inflammation, oxidative stress, lipid accumulation, and energy metabolism. As the links between the complement system and AMD are becoming clearer, many innovative therapeutic attempts targeted at complement components emerge in the therapy of AMD. To date, there is still a lack of up-to-date and comprehensive papers on the involvement of the complement system in the pathogenesis of AMD and the research actuality of complement inhibitors in the treatment of dry AMD versus wet AMD, even though there have been a number of studies and review articles [9, 10]. This review will focus on the application of complement inhibitors in dry AMD and wet AMD, covering the emerging data on clinical trials, the genetic and molecular associations behind, and the challenges and prospects before this treatment become a formal therapy in the future.

## 2. Complement System and AMD

### 2.1. Overview of Complement System

The complement system, first described as an auxiliary system by Jules Bordet and Paul Ehrlich in the late 19th century, is a highly regulated protein network which can be activated in a cascaded manner, and acts at the interface of innate and adaptive immunity [11]. To some extent, the complement system is a double-edged sword. Under normal circumstances, it is the most significant protective mechanism, playing a key role in tissue homeostasis and pathogen immunosurveillance in the body [12]. However, under abnormal circumstances, its impaired regulation or dysfunction can be the main cause of a variety of acute and chronic disorders like atypical hemolytic uremic syndrome, paroxysmal nocturnal hemoglobinuria, C3 glomerulopathy, and AMD.

**Figure 1:**
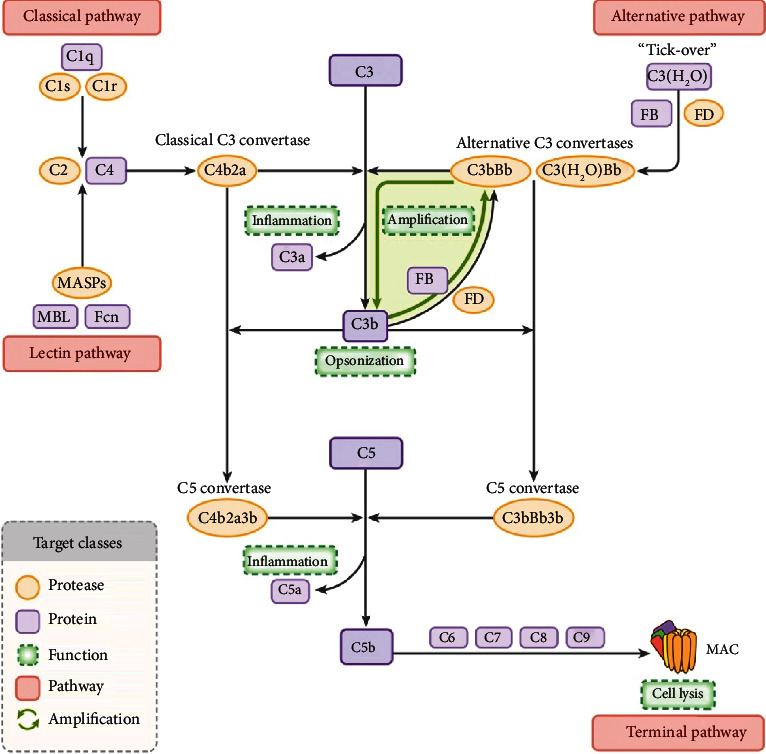
The complement cascade can be activated in three proteolytic pathways: classical pathway (CP), lectin pathway(LP), and alternative pathway (AP). All of them will converge on C3 and C5 and bring about the terminal pathway ultimately, the formation of the membrane attack complex (MAC), which can lead to cell lysis.

Generally, the complement cascade is activated via three different proteolytic pathways: the classical pathway (CP), lectin pathway (LP), and alternative pathway (AP) [13] (Figure 1). Specifically, although CP commonly responds to antigen-antibody complexes and LP is started by mannose-binding lectin (MBL) and identification of the polysaccharide or glycoprotein motif on the damaged cell surface [14], both of them will produce a common membrane-bound C4b2a (classical C3 convertase) afterwards. Furthermore, AP can be activated in two ways: by spontaneous hydrolysis of C3 into C3(H_2_O) in the fluid phase, also known as “tick-over,” and by C4b2a (C3 convertase) to cleave C3 into C3a and C3b in the solid phase [6, 15]. Subsequently, both C3(H_2_O) and C3b can be bonded by factor B (FB) and factor D (FD) to form C3(H_2_O)Bb and C3bBb, respectively. Of note, AP has a positive feedback on the generation of ample amounts of C3b, which is regarded as the amplification loop between C3bBb and C3b for the other two pathways (Figure 1). Consequently, the two sourced C3 convertases will drive C3b production further in tissues to opsonize the pathogen.

In the final stage, the convergence of the C3 convertase and additional C3b results in the formation of membrane-bound enzyme complexes (C5 convertases), which integrate all processes into a common terminal pathway. The C5 convertase will cleave C5 into C5a and C5b afterwards. Furthermore, owning a new short-lived binding site, C5b sequentially recruits C6, C7, C8, and C9 to form the terminal complex C5b-9, also referred to as the membrane attack complex (MAC). Formation of MAC will lead to a pore in the target cell membrane and ultimately causes cell lysis and death, which can be downregulated by complement inhibitors like CD59 [16, 17].

CD59 is a naturally existing inhibitor of MAC formation that functions by binding to the terminal complement protein complex, preventing C9 molecules from binding to the cell membrane, and thereby forming pores [18]. The complement system can be regulated by different classes of drugs; all levels of the cascade can be affected. Notably, regardless of the initiating pathway, AP accounts for about 80–90% of the activation of the terminal pathway [19]. Thus, overactivation of the complement system in the AP pathway is one of the major drivers of many systemic and organ specific diseases [20, 21].

### 2.2. AMD as a Multifactorial Disease

As an incredibly complex, multifactorial disease, AMD is driven by a combination of natural aging, unhealthy lifestyle, and genetic predisposition. With increasing age, mitochondria within the RPE cells of AMD patients decrease in size and number and produce more waste products [22, 23], while choroidal elasticity weakens and the ability to process waste products is diminished [24, 25], ultimately leading to impairment of Bruch's membrane function, which triggers a vicious cycle of continuous debris deposition that is considered to be the formation of drusen [26, 27]. In recent years, smoking and nutritional intake have been identified as important risk factors for AMD. The risk of AMD from smoking is dose-dependent, with a reduced risk of developing AMD after quitting, whereas smoking can increase the risk two to fourfold [28, 29]. In terms of diet, a hyperglycemic diet is an important risk factor for AMD [30, 31], whereas a “Mediterranean diet” rich in vitamins and carotenoids may reduce the risk of AMD [32–34]. In addition, fish intake has been shown to have a protective effect against AMD [35]. With regard to genetic variation, genome-wide association studies (GWAS) have established 35 discrete loci with more than fifty independently linked genetic variants, a large proportion of which are related to the complement system [36, 37]. In the following two sections, we will describe the involvement of the complement system in the development and progression of AMD at genetic and molecular levels, respectively.

### 2.3. Genetic Studies of Complement System in AMD

The relevance between AMD and complement system has been revealed in numerous researches since the 1980s, both locally and systemically: (a) the existence of complement components in drusen, for example, C3a and complement factor H (FH) [27, 38–42]; (b) the elevated levels of systemic [43–46] and local (aqueous humor samples and vitreous humor) complement proteins in AMD patients [20, 47, 48]; (c) the decreased levels of regulatory complement proteins in the eyes of AMD patients [49, 50]; and (d) the increased level of membrane attack complex (a terminal complex of complement cascades) in the retinas of AMD patients [51]. Until 2005, genetic evidence of the complement system in the etiology of AMD was first demonstrated in GWAS [5], which indicated that a common single nucleotide polymorphism (SNP) in the complement FH gene is related to an increased risk of AMD. Subsequently, a lot of studies revealed that other variations in multiple complement genes, such as complement FI, C3, FB, FD, and C9, can increase the risk of AMD as well [37, 52–55].

As an important inhibitor of the complement system, complement FH can not only compete with FB to bind C3b, accelerating the dissociation of the alternative system C3 convertase (C3bBb) resultingly, but also act as a cofactor for facilitating the FI-mediated C3b inactivation [56, 57]. Genetic variations in FH can reduce the effectiveness of modulation in the complement cascade activity, leading to larger precipitation of complement component found in drusen [58]. Up to now, there are a total of 160 coding variants in the FH gene discovered in AMD, among which 16 are nonsense changes, five are frameshift changes, and 139 are missense changes in the FH protein [59]. Nevertheless, their effect needs to be further studied for the vast majority of coding variants in FH. It is worth mentioning that 42% Europeans are heterozygous for the Y402H variant, which is regarded to be consistently associated with the occurrence and progression of AMD. It is reported that homozygous individuals have about 7-fold greater odds of relationship with AMD, while heterozygotes own 2- to 3-fold greater odds of relationship with the disease [15]. The inhibitory effect of FH on the complement system is assumed to be reduced owing to the decreased binding affinity of many complement components of the damaged retina led by the Y402H variant, which further results in excessive chronic local inflammation to occur [49, 60, 61]. Despite extremely rare, R1210C has a closer association with AMD than Y402H [62]. It was presumed that the binding of R1210C mutant to albumin can bring about the loss of function in complement FH [62]. Further studies on functional analysis revealed that R1210C variant decreased binding to C3b, C3d, and heparin to increase activity of the C3 convertase [63–65]. Other rare variants in complement FH SNPs, including R53C and D90G, have also been noted to influence AMD [62, 66, 67].

As mentioned above, C3 is the key component among all complement pathways. Any functional changes in C3 can directly influence the downriver cascade. Therefore, C3 gene is another important genetic variant in AMD. R102G variant is the most commonly happening mutation in the C3 gene of AMD patients [59]. The polymorphism of R102G in C3 reduces the activity of FH as a cofactor that mediates C3b cleavage by FI. That is, it increases the activation of the AP by prolonging the lifespan of the convertase [68]. Moreover, R80G is a common SNP associated with an ascending risk of AMD [69, 70]. Furthermore, total 91 rare variants in C3 gene are demonstrated in AMD [71, 72]. One of these rare variants is K155Q, positively related to AMD risk, which is located very close to the FH-binding site [73, 74]. Thus, the allele of K155Q can increase the resistance to proteolytic inactivation via FH and FI [75].

The complement FI gene contains a serine protease domain which is the cause of cleaving and inactivating C3b and C4b to regulate complement activation. To date, there are 110 variants in the complement FI gene discovered in AMD patients, among which nine are nonsense changes, one is frameshift change, and 100 are missense changes in the FI protein [59]. A case-control association study for advanced AMD reported that rs10033900A is a common variant near the FI gene, showing the strong association with AMD. A cohort study that included 2,493 advanced AMD patients indicated that 7.8% of AMD cases compared to 2.3% of controls (odds ratio 3.6) are carriers of rare missense changes in the FI variants [73].

Both serine proteases FB and FD have significant roles in the formation and activity of the C3 convertase in AP [6, 76]. Of note, both L9H and R32Q mutations of factor B are considered to have a high protective effect on the development of AMD, although this protective effect may be mediated by FB mutations [77, 78]. Subsequent study has shown that FB fragments are similar to FH levels found in drusen, while R32Q mutations decreased the formation of convertase [79]. Cleaving FB into Ba and Bb fragments, FD is regarded as the rate-limiting enzyme of AP [6, 76]. A small case-control series showed that rs3826945 (a FD gene SNP) is considered positively linked to AMD risk as well [80].

C9 is the most downstream component of the terminal complement pathway. A remarkable decreasing of C9 can affect the production of MAC and finally decrease the cytolytic activity [81–83]. At present, there are 37 variants in the C9 gene discovered in AMD, among which six are nonsense changes, three are frameshift changes, and 28 are missense changes in the C9 protein [59]. The relationship between AMD and a P167S allele in C9 gene has been discovered by genotyping 5,115 independent samples [73]. In another small sample study, the R95X variant in the C9 gene was negatively linked with the risk of AMD [84].

Taken together, on the one hand, these findings suggest that genetic variants that hinder the negative regulation of the complement system promote the development of AMD. Notably, it has been suggested that the additive effect of risk variants leads to an aggregated risk of disease [85]. On the other hand, many variants found in genes related to the complement system highlight the importance of this immunologic pathway in AMD etiology, which can provide potential targets for complement therapy [86–91].

### 2.4. Molecular Studies of Complement System in AMD

While genetic studies have been somewhat successful in determining the risk of the complement system for the development of AMD, the understanding of how these variants drive AMD at the molecular level remains largely incomplete. Research on the molecular mechanisms of the complement system will not only provide a deeper understanding of AMD but will also contribute to the discovery of future drug treatments. To date, molecular studies of the complement system in AMD have focused on following main areas: chronic inflammation, oxidative stress, lipid accumulation, and energy metabolism.

The most notable consequence of complement activation is that it can mediate the recruitment and activation of immune cells, such as microglia, monocytes/macrophages, lymphocytes, and mast cells, through the release of complement components C3a and C5a [92, 93]. In addition to this, complement activation stimulates surrounding RPE cells to secrete a range of inflammatory factors, for instance, monocyte chemotactic protein 1, interleukin 6, and interleukin 8 [94]. Chronic inflammation is a typical ocular change in AMD. Due to the significant role of the complement system in the inflammatory response to AMD, complement inhibitors targeting C3 and C5 are developed for treatment, and the relevant clinical trials will be described in the next section.

Drusen is a characteristic fundus change in AMD, and it is now known that lipids are a major component of drusen [26]. Indeed, high-risk polymorphisms in the complement gene and dysregulation of the complement system are both related to the local or systemic lipid accumulation. Metabolomic studies performed on plasma/serum from AMD patients have shown a strong correlation between increased high-density lipoprotein (HDL) levels and reduced very low-density lipoprotein (VLDL) and amino acid levels, which are linked with the excessive activation of the complement system. It is now well established that oxidized low-density lipoprotein (LDL) results in the upregulation of C-C motif chemokine receptor 2, interleukin 8, tumor necrosis factor, and VEGF and that binding of FH to these oxidized LDLs attenuates inflammation [95–97]. However, it remains unknown whether the presence of FH in HDL particles has a protective effect [21].

In recent years, several studies have exposed RPE cells to different types of stress stimuli separately, measuring and analyzing the levels of complement proteins and complement factors. For example, extracellular levels and genetic levels of C3 were significantly improved when human adult retinal pigment epithelial cells (ARPE-19) were exposed to H_2_O_2_, smoke extracts, and lipid oxidation products [98–102]. In addition, FH has been found to in human plasma in two distinct redox forms. The reduced form is higher in patients with early AMD and protects ARPE19 cells from oxidative damage, whereas the oxidized form is higher in patients with advanced AMD and can effectively mediate FI to accelerate C3 cleavage [103].

Recently, imbalances in energy metabolism within RPE cells have been considered as an important aspect of AMD pathology. An experimental animal study showed abnormally large mitochondria, reduced levels of mitochondrial DNA, and decreased ATP production in photoreceptors and RPE cells in FH knockout mice [104, 105]. This suggests that dysregulation of the complement system can reduce cellular energy metabolism by affecting the structure and function of mitochondria. Furthermore, a link between the complement system and autophagy-lysosomes has recently been demonstrated [106].

## 3. Complement Inhibitors and AMD

### 3.1. Overview of Complement Therapy in AMD

The current treatment strategy for AMD is not optimistic. Although anti-VEGF injection is an effective treatment option for wet AMD, there is still a proportion of patients who are not sensitive to the drug. In addition, even with monthly anti-VEGF therapy, GA still remains an inevitable long-term progressive outcome for a majority of patients with wet AMD [107, 108]. However, there are no approved treatments or effective approaches for dry AMD. Therefore, the exploration of treatments for AMD remains an important area of research for ophthalmologists.

In the past decades, as the first FDA-approved complement inhibitor, eculizumab (C5 inhibitor) is used to treat the hemolytic disorder, paroxysmal nocturnal hemoglobinuria (PNH), which remarks an important milestone in complement drug discovery [109]. Since then, complement inhibitors have been receiving more attention gradually. To date, more than 14 complement inhibitors have been generated for core complement components (C3, C5) and complement regulators (FD, FI, etc.), and a total of nearly 40 clinical trials of these complement inhibitors have been completed or are under way in AMD. The main outcome indicators in these clinical trials are changes in the size of the GA lesion or improvements in visual function. Given the urgent need for the treatment of dry AMD, the majority of these clinical trials have focused on GA [87]. Here, we will classify the clinical trials according to dry AMD and wet AMD and update the progress of them, respectively.

### 3.2. Complement Inhibitors in Dry AMD

Although numerous studies have explored the etiology and pathogenesis of dry AMD, there is still a lack of an effective strategy for the treatment. Therefore, compared with wet AMD, it is more urgent to devise an efficiently and feasible treatment for dry AMD. According to the statistics, in dry AMD, there are at least 13 different complement inhibitors which have been applied in nearly 30 different clinical trials (Table 1). Next, we will summarize them one by one according to the common targets of different drugs.

Targeting at C3, three therapeutic drugs have been devised for dry AMD so far. Firstly, POT-4, also referred to as AL-78898A, is a compstatin derivative, which is also the first complement inhibitor to be employed in patients. Preliminary results have shown that intravitreal POT-4 is safe and well-tolerated (NCT00473928). However, the trial was terminated prematurely due to the deposit formation of drugs in the eye of GA in phase II trial (NCT01603043). The second is APL-2, also known as pegcetacoplan, another peptide compstatin analogue, which reduces the GA growth rate by 29% monthly and by 20% every other month (EOM) in phase II trial, compared with the sham treatment group (NCT02503332) [110]. Of note, the neovascularization of dry AMD was reported more frequently in APL-2-treated eyes (20.9%, 8.9%, and 1.2% in monthly groups, EOM groups, and sham groups, respectively). Phase III clinical trials of APL-2 are under way with the same dosage regimen, but the difference is that the duration of treatment has been extended to 2 years (NCT03525613). Moreover, an extension study to assess the long-term safety and efficacy is currently under way (NCT03777332). Lastly, as a humanized IgG1 monoclonal antibody engineered to potently inhibit the activity of C3, the performance of NGM621 is safe and well-tolerated in phase I trials. Further studies will be conducted with doses of 15 mg injected every 4 weeks or every 8 weeks in phase II trial (NCT04465955).

Targeting at C5, there are three therapeutic drugs explored for GA up to now. First, as the first FDA-approved complement inhibitor, eculizumab was safe and well-tolerated in the body through 6 months. However, eculizumab did not significantly reduce the growth rate of GA or the volume of drusen by intravenous fluid (NCT00935883) [111, 112]. Secondly, as a monoclonal C5 inhibitor, LFG316 (tesidolumab) has passed the safety evaluation but still inhibits the progression of GA lesions ineffectively in phase II trials (NCT01527500). Finally, Zimura, also known as avacincaptad pegol, is a pegylated RNA aptamer, which is a specific and potent inhibitor of C5. Compared with the sham group, intravitreal administration of Zimura 2 mg and 4 mg dose groups can reduce 27.4% and 27.8% of mean rate of GA growth over one year, respectively, which is of statistical significance in the phase II/III trials (NCT02686658) [113]. In addition, treatment with Zimura showed the increased dose-dependent risk of CNV in treated eyes: 2.7%, 11.9%, and 15.7% in sham, 2 mg dose, and 4 mg dose cohorts, respectively. Currently, focusing on the safety and efficacy of Zimura in slowing down the rate of GA growth, phase III trials of confirmation are under way (NCT04435366).

On the one hand, several inhibiting activators have been targeted in clinical trials, such as FD, properdin, and FB. As is known to all, complement FD is a pivotal activator of complement AP, which is the target of lampalizumab, a selective monoclonal complement FD inhibitor. In phase II, a positive result indicated that monthly lampalizumab treatment demonstrates a 20% reduction in the progression of GA lesion area (NCT02288559) [114]. However, the greatest studies of GA have shown that lampalizumab does not decrease the enlargement of GA over 12 months in phase III, and this trial terminated afterwards (NCT02247479, NCT02247531) [115, 116]. On top of that, CLG561, a fully human antibody Fab, can neutralize properdin to prevent the formation of early and late activation products. In phase I trials, single intravitreal doses of CLG561 are safe and well-tolerated (NCT01835015). Unfortunately, CLG561 were evaluated as a monotherapy or in combination with LFG316 (a C5 inhibitor), and there is no effect on the change of GA lesions in the phase II clinical trial (NCT02515942). Besides, as a novel drug, IONIS-FB-LRx is a ligand-conjugated antisense inhibitor, which can be administered subcutaneously for systemic reduction in circulating FB levels, with a potential to diminish the systemic overactivity of the alternative pathway. It is being assessed in a phase II trial for dry AMD patients. In phase I trials, subcutaneous injection of IONIS-FB-LRx has been proved safe in 54 healthy volunteers. Based on this, a phase II trial has been initiated to evaluate the effectiveness of IONIS-FB-LRx in GA patients (NCT03815825).

On the other hand, a few supplementing regulators of the complement system have been targeted in clinical trials, for example, CD59, FI, and FH. Firstly, AAVCAGsCD59, also named as HMR59, uses an AAV2 gene therapy and is designed to induce the generation of a soluble CD59 protein, which binds the incomplete MAC and blocks the binding of the C9 protein required to complete the complex [117, 118]. In phase I clinical trials, AAVCAGsCD59 is delivered intravitreally and well-tolerated (NCT03144999). Of note, while the phase I trials are not designed to judge efficacy, it is encouraging that 9 of 11 cases demonstrated a slower rate of GA growth, which promotes it to be tested for the efficacy of GA in phase II trials (NCT04358471).

Furthermore, two clinical trials based on genotype to select patients have been performed. Specifically, before the inclusion in the trials of GT005 and GEM103, patients are chosen for carrying risk gene variants in complement FI and FH. Similar to AAVCAGsCD59, GT005 uses an AAV vector and is designed to the supply of FI protein to the treated eye. Nowadays, phase I/II trials of GT005 in patients with GA are assessing its effectiveness in the UK. As therapy via subretinal injection, GT005 is currently in phase II clinical trials (NCT03846193). Moreover, gene therapy provides the potential for a single injection lasting for 1 year or longer, which offers the benefit of requiring fewer injections to treat AMD patients should the drug show efficacy and safety in future trials. As an endogenous human FH protein, GEM103 is administered via intravitreal injection. The safety and tolerability of GEM103 has been shown in phase I trials. Ocular pharmacokinetic and pharmacodynamic effects were assessed in aqueous humor samples for two months after treatment, showing that sustained supranormal levels of drug concentrations were achieved after treatment.

Finally, ANX007 is a monoclonal antibody antigen-binding fragment (Fab), which can potently bind to C1q to inhibit the activation of CP, including C3 and C5. ANX007 has been tested in patients with primary open-angle glaucoma, and the safety is confirmed, and it is well-tolerated in the eye. At present, ANX007 is used in investigating the safety and efficacy of intravitreal injections in patients with GA in phase II (NCT04656561).

### 3.3. Complement Inhibitors in Wet AMD

Since the approval of the first injectable anti-VEGF drug in 2004, intravitreal injections of anti-VEGF drugs have become the first-line treatment for wet AMD patients to suppress CNV and improve visional function currently [119]. A recent systematic literature review demonstrated that the introduction of anti-VEGF therapy in clinical practice has been associated with a significant reduction in the prevalence of blindness [120]. Nevertheless, this strategy still has a few limitations. Rofagha et al. studied the outcome of about 7-year ranibizumab-treated patients and found only one-third of the outcomes to be good with a visual decline observed in half of the patients [121]. Thus, there are still vast unmet clinical demands in wet AMD; exploring new treatment strategies is still needed. Up to now, there are 6 complement inhibitors which have been investigated in 10 clinical trials in wet AMD (Table 2).

Targeting at C3, two therapeutic drugs have been devised for wet AMD patients so far. The intravitreal delivery of POT-4 was compared to anti-VEGF in patients with active wet AMD in a phase II trial. As a result, the advantage of POT-4 over anti-VEGF drugs could not be demonstrated. Instead, the AMD participants treated with POT-4 had increased retinal thickness at week 4, while patients injected with anti-VEGF had decreased retinal thickness. POT-4 was unable to reduce central retinal thickness 12-week posttreatment, as seen with ranibizumab (NCT01157065). The other is APL-2, which is delivered intravitreally and well-tolerated in phase I trials (NCT02461771). APL-2 were evaluated in patients with active wet AMD on anti-VEGF treatment in phase II as well. However, this clinical trial was terminated due to the ineffectiveness of treatment when 17 participants were collected (NCT03465709).

Targeting at C5, there are two therapeutic agents that have been explored in wet AMD to date. LFG316 drug was also assessed for efficacy in phase II trials, unfortunately, neither reduction in the number of anti-VEGF injections (NCT01535950) nor improvement in BCVA and macular thickness (NCT01624636). Zimura combination therapy with ranibizumab for wet AMD was safe and well-tolerated after 6 months of treatment in a phase II trial as well (NCT00709527, NCT03362190). Of note, in patients receiving monthly Zimura 2 mg in combination with anti-VEGF 0.5 mg, approximately 60% achieved visual acuity improvement greater than or equal to three lines, which was better than the results of anti-VEGF monotherapy.

Moreover, two new drugs are undergoing phase I clinical trials. Firstly, intravitreal delivery of AAVCAGsCD59 is currently in phase I clinical evaluation (NCT03585556). Previous studies have shown that subretinal injection of AAVCAGsCD59 attenuated the formation of laser-induced CNV by around 60% in mice [122]. In phase I trials, all new-onset wet AMD patients received anti-VEGF treatment at day 0 and subsequently accepted intravitreal AAVCAGsCD59 for one week. The combination of gene therapy and anti-VEGF treatment is regarded as a promising approach to wet AMD patients [123]. The other is IBI302, which is globally the first bispecific recombinant fully human fusion protein. Its N-terminus binds to VEGF family, thereby blocking the VEGF-mediated signaling pathway. Its C-terminus binds specifically to C3b and C4b, inhibiting the activation of the complement cascade of CP and AP and reducing the complement-mediated inflammatory response, leading to the treatment of wet AMD. The additional targets added to IBI302 could provide larger clinical benefit compared to anti-VEGF drugs. The study was presented at the 2020 American Academy of Ophthalmology (AAO). To date, 31 subjects have participated in phase I clinical trials, and the safety and tolerability of IBI302 are well (NCT03814291). The results from these clinical trials will provide insight into the potential of complement inhibition and combination therapy in the future [124].

## 4. Challenges and Prospects

Nowadays, despite complement inhibitors seeming promising in theory or even in vitro studies, the majority of clinical trials aiming at this class of drugs in AMD prove to have a modest effect, which might be relevant to the following five reasons.

First of all, the selective permeability of drugs may be a factor that cannot be ignored. Indeed, our eye is a highly protected organ and the blood-ocular barrier effectively shields it from macromolecules. In general, the molecule cannot penetrate the eye when its size exceeds 76.5 ± 1.5 kDa [125]. FD has been shown to diffuse through Bruch's membrane, whereas most of the complement components do not, such as FH, FI, FB, and C3/C3b [126]. Thus, it has been speculated that the failure of eculizumab is because of its large molecular size (148 kDa) [127], and the results of this trial might have been altered with better drug design and improvements in the drug delivery system.

Secondly, the timing of intervention may be an important reason. Almost all complement inhibitors focus on the treatment of advanced stage in dry AMD, but the results are barely succeeded. A possible explanation may be that when the disease has evolved to the stage of GA, irreversible structural and functional damage of the retina has already occurred at this point [128]. Not surprisingly, even though the complement system is the significant factor in the pathogenesis of AMD, the clinical treatment efficacy of complement inhibitors is likely to be minimal at that time. Consequently, it may be a new breakthrough point to initiate the intervention at an earlier stage in the following study [127].

Thirdly, the inconsistency between the target of drug therapy and the dominant cause of patients may be another significant factor. Given that AMD is a multifactorial disorder, complement inhibitors that act on a specific target may be only suitable for patients who carry a genetic risk factor predisposing to overactivation of complement at that specific target, while not applied to all AMD patients. For instance, patients who are homozygous for the FH 402H variant would have a 7.4-fold greater risk from AMD in elderly people than those who are heterozygous for the 402H variant [53]. Therefore, correct selection of patients who have genetically supported targets to conduct drug development accordingly and clinical trials may increase the odds of success in clinical drug development [129].

Fourthly, the frequency of intervention might be a potential reason as well. For example, while lampalizumab demonstrated the potential to improve the progression of GA in phase II trials, it was unable to reproduce such outcome and efficacy in phase III trials. A probable cause may be the lower frequency of medical treatment, every 2 or 4 weeks in phase II while every 4 or 6 weeks in phase III, reducing the effectiveness of treatment [115, 130]. In this way, the medical influence brought by different frequencies of complement therapy is of significant value and deserves to be studied further in the future.

Finally, the limitations of assessment methods may also be a relevant factor. At present, in most clinical trials, the checking means of evaluating the curative effect is fundus autofluorescence (FAF). However, FAF has poor susceptibility to media opacities because of the macular pigment that absorbs blue light, thereby causing difficulty in imaging the fovea [131]. Therefore, the accuracy of the efficacy assessment for those patients will also be affected. Moreover, quantitative evaluation of efficacy remains to be a challenge [81]. If more accurate and sophisticated detection tools could be devised, then the effects of complement inhibitors on patients with AMD can be observed more creditably and comprehensively in future studies.

Although complement therapy is confronted with multiple challenges currently, its therapeutic future remains promising. Both APL-2 and Zimura have demonstrated modest success in inhibiting the progress of GA. With the improvement of visual acuity as an ideal goal, both of them have passed phase II clinical trials and are undergoing further verification in phase III clinical trials. Of note, the inhibition of C3 through APL-2 induced neovascularization, prompting a tendency to convert dry AMD to wet AMD. Even though there remain unrevealed risks of neovascularization in dry AMD, treatment with anti-VEGF after conversion to wet AMD is also a possible treatment strategy for this group of patients [81]. In addition, the performance of several new drugs and combination therapies, such as AAVCAGsCD59, applied in phase II trials is also promising.

## 5. Conclusion

The complement system indeed plays a remarkable role in the development and progression of AMD. Targeting at different complement components, many clinical trials of complement inhibitors have been conducted or are ongoing in AMD. While most complement inhibitors fail to demonstrate the potential, APL-2 and Zimura are effective in inhibiting the rate of GA progression in phase II clinical trials, and both of them successfully entered phase III trials. Furthermore, the combination of Zimura and ranibizumab resulted in a significant improvement in visual acuity in patients with wet AMD at phase II clinical trials compared to ranibizumab monotherapy. Of note, the performance of Zimura in both dry AMD and wet AMD is quite encouraging. However, whether Zimura is expected to be a new drug for dry AMD is pending the results of phase III clinical trials. Overall, complement inhibitors have shown potential in the future treatment of AMD and deserve to be explored further.

## Figures and Tables

**Table 1:** Summary of complement inhibitors in clinical trials for dry AMD.

Target	Drug (sponsor)	Administration	Phase	Design	Sample size (*n*=)	Primary outcome measure	Status	First posted	Trial number	Clinical outcome
	POT-4 (Alcon)	Intravitreal	II	Monthly vs. sham	10	GA area change at month 12 by FAF	Terminated	2012	NCT01603043	Drug deposit formation
	APL-2 (Apellis)	Intravitreal	II	Monthly vs. EOM vs. sham	246	Square root of GA area change at 12 months	Completed	2015	NCT02503332	29% reduction in GA lesion growth
C3	APL-2 (Apellis)	Intravitreal	III	Monthly vs. EOM vs. sham	600 (estimated)	GA area change at month 12 by FAF	Ongoing	2018	NCT03525613	—
	APL-2 (Apellis)	Intravitreal	III	Monthly vs. EOM vs. sham	1200 (estimated)	Percentage of adverse events at month 36	Ongoing	2021	NCT03777332	—

	NGM621 (NGM bio.)	Intravitreal	II	Every 4 weeks vs. 8 weeks vs. sham	240 (estimated)	GA area change at week 48 by FAF	Ongoing	2020	NCT04465955	—
	Eculizumab (Alexion)	Intravenous	II	Low dose vs. high dose	60	Growth of GA and decrease in drusen volume	Completed	2009	NCT00935883	Lack of efficacy
	LFG316 (Novartis)	Intravitreal	I	SAD	24	Safety and tolerability	Completed	2010	NCT01255462	Safe and well-tolerated
	LFG316 (Novartis)	Intravitreal	II	Low dose vs. high dose vs. sham	158	GA area change at day 505 by FAF	Completed	2012	NCT01527500	Lack of efficacy

C5	ARC1905 (Ophthotech)	Intravitreal	I	Low dose vs. high dose	47	Safety and tolerability	Completed	2009	NCT00950638	Safe and well-tolerated
	ARC1905 (Ophthotech)	Intravitreal	II/III	Low dose vs. high dose vs. sham	286	GA area change at month 12 by FAF	Completed	2016	NCT02686658	27% reduction in GA lesion growth
	ARC1905 (Ophthotech)	Intravitreal	III	Monthly vs. EOM vs. sham	400 (estimated)	Mean rate of GA change at month 12 by FAF	Ongoing	2020	NCT04435366	—

	Lampalizumab (Roche)	Intravitreal	Ia	SAD	18	Safety and tolerability	Completed	2009	NCT00973011	Safe and well-tolerated
FD	Lampalizumab (Roche)	Intravitreal	II	Every 2 weeks vs. 4 weeks vs. sham	96	GA area change at week 24 by FAF	Completed	2014	NCT02288559	20% reduction in GA lesion area

	Lampalizumab (Roche)	Intravitreal	II	Monthly vs. EOM vs. sham	159	Percentage of adverse events	Terminated	2012	NCT01229215 NCT01602120	Lack of efficacy
FD	Lampalizumab (Roche)	Intravitreal	III	Every 4 weeks vs. 6 weeks vs. sham	906	GA area change at week 48 by FAF	Terminated	2014	NCT02247479 NCT02247531	Lack of efficacy
	Lampalizumab (Roche)	Intravitreal	III	Every 4 weeks vs. every 6 weeks	994	Percentage of adverse events at week 96	Terminated	2016	NCT02745119	Lack of efficacy

	CLG561 (Novartis)	Intravitreal	I	SAD (5 dose levels)	50	Safety and tolerability	Completed	2013	NCT01835015	Safe and well-tolerated
Properdin	CLG561 (Novartis)	Intravitreal	II	CLG561 vs. CLG561+LFG316 vs. sham	114	Percentage of adverse events, GA area change at day 337 by FAF	Completed	2015	NCT02515942	Lack of efficacy

FB	IONIS-FB-LRx (Lonis)	Subcutaneous	II	IONIS-FB-LRx vs. placebo	330 (estimated)	GA area change at week 49 by retinal imaging	Ongoing	2019	NCT03815825	—

	AAVCAGsCD59 (Hemera)	Intravitreal	I	SAD (3 dose levels)	17	Percentage of adverse events at week 26	Completed	2017	NCT03144999	Safe and well-tolerated
CD59	AAVCAGsCD59 (Hemera)	Intravitreal	II	Low dose vs. high dose vs. sham	132 (estimated)	GA area change at month 24 by FAF	Ongoing	2020	NCT04358471	—

	GT005 (gyroscope)	Subretinal	I/II	SAD (3 dose levels)	45 (estimated)	Percentage of adverse events at week 48	Ongoing	2019	NCT03846193	—
FI	GT005 (gyroscope)	Subretinal	II	Low dose vs. high dose vs. sham	180 (estimated)	GA area change at week 48 by FAF	Ongoing	2020	NCT04437368 NCT04566445	—

FH	GEM103 (Gemini)	Intravitreal	I	SAD (4 dose levels)	12	Safety and tolerability	Completed	2020	NCT04246866	Safe and well-tolerated
	GEM103 (Gemini)	Intravitreal	II	GEM103 vs. sham	45 (estimated)	Percentage of adverse events at month 18	Ongoing	2020	NCT04643886	—

C1q	ANX007 (Annexon)	Intravitreal	II	Monthly vs. EOM vs. sham	240 (estimated)	GA area change at month 12 by FAF	Ongoing	2020	NCT04656561	—

FD: factor D; FB: factor B; FI: factor I; FH: factor H; EOM: every other month; SAD: single ascending dose; GA: geographic atrophy; FAF: fundus autofluorescence.

**Table 2:** Summary of complement inhibitors in clinical trials for wet AMD.

Target	Drug (sponsor)	Administration	Phase	Design	Sample size (*n*=)	Outcome measure	Status	First posted	Trial number	Clinical outcome
	POT-4 (Potentia)	Intravitreal	I	SAD	27	Safety and tolerability	Completed	2007	NCT00473928	Safe and well-tolerated
	POT-4 (Potentia)	Intravitreal	II	POT-4 vs. ranibizumab	99	Central subfield retinal thickness at week 4	Completed	2010	NCT01157065	Lack of efficacy
C3	APL-2 (Apellis)	Intravitreal	I	SAD	13	Safety and tolerability	Completed	2015	NCT02461771	Safe and well-tolerated

	APL-2 (Apellis)	Intravitreal	Ib/II	APL-2	17	Percentage of adverse events at 1 year	Terminated	2018	NCT03465709	Lack of efficacy
	LFG316 (Novartis)	Intravitreal	II	LFG316 vs. sham	43	Number of anti-VEGF treatments	Completed	2012	NCT01535950	Lack of efficacy
	LFG316 (Novartis)	Intravenous	II	Low dose vs. high dose vs. placebo	1	Safety and tolerability	Terminated	2012	NCT01624636	Lack of efficacy
C5	ARC1905 (Ophthotech)	Intravitreal	I	ARC1905+ranibizumab vs. ranibizumab	60	Safety and tolerability	Completed	2008	NCT00709527	Safe and well-tolerated
	ARC1905 (Ophthotech)	Intravitreal	IIa	ARC1905 (3 dose levels)+ranibizumab	65	Percentage of adverse events at month 6	Completed	2017	NCT03362190	No adverse events

CD59	AAVCAGsCD59 (Hemera)	Intravitreal	I	AAVCAGsCD59+anti-VEGF	25 (estimated)	Number of anti-VEGF treatments	Ongoing	2018	NCT03585556	—

C3b C4b	IBI302 (Innovent)	Intravitreal	I	IBI302 (6 dose levels)	180 (estimated)	Safety and tolerability	Ongoing	2019	NCT03814291	—
